# SMARThealth PRegnancy And Mental Health study: protocol for a situational analysis of perinatal mental health in women living in rural India

**DOI:** 10.3389/fgwh.2023.1143880

**Published:** 2023-07-27

**Authors:** Nicole Votruba, Devarsetty Praveen, Lucy Mellers, Eldho Rajan, Sudhir Raj Thout, Varun Arora, Yogender Malik, Aditya Kashyap, Sreya Majumdar, Jane Hirst, Pallab K. Maulik

**Affiliations:** ^1^ Department of Women’s & Reproductive Health, University of Oxford, Oxford, United Kingdom; ^2^ The George Institute for Global Health, Imperial College London, London, United Kingdom; ^3^ The George Institute for Global Health India, New Delhi, India; ^4^ The George Institute for Global Health, University of New South Wales, Sydney, NSW, Australia; ^5^ Post Graduate Institute of Medical Science, Rohtak, India; ^6^ Department of Psychiatry, Institute of Mental Health (IMH), University of Health Sciences PGIMS, Rohtak, India; ^7^ SVS Institute of Neurosciences, Government Medical College, Siddipet, India

**Keywords:** perinatal mental health, maternal mental health, mental health in pregnancy, maternal depression and anxiety, stigma & discrimination, India, situational analysis, low- and middle-income countries

## Abstract

**Introduction:**

The situation for women experiencing mental health problems during pregnancy and postpartum in rural India is critical: a high burden of disease, a high estimated number of women are undiagnosed and untreated with mental health problems, a substantial gap in research on women's perinatal health, and severe stigma and discrimination. The SMARThealth Pregnancy study is a cluster randomised trial using a digital intervention to identify and manage anaemia, hypertension, and diabetes in the first year after birth in rural India. Within this study, the SMARThealth Pregnancy and Mental Health (PRAMH) study is a situational analysis to understand mental health problems during pregnancy and in the first year following birth in this population.

**Methods/design:**

This situational analysis aims to analyse and to assess the context of perinatal mental health, health services, barriers, facilitators, and gaps in Siddipet district of Telangana state in India, to develop an implementation framework for a future intervention. A tested, standardised situational analysis tool will be adapted and applied to perinatal mental health in rural India. A desktop and policy review will be conducted to identify and analyse relevant mental health and pregnancy care policies at the national and state levels. We will conduct in-depth interviews with policymakers, planners, mental health professionals and other experts in perinatal mental health (*n* = 10–15). We will also conduct focus group discussions with key stakeholders, including women with perinatal mental health problems, their families and carers, and community health workers (*n* = 24–40). A theory of change workshop with key stakeholders will be conducted which will also serve as a priority setting exercise, and will clarify challenges and opportunities, priorities, and objectives for a pilot intervention study. The analysis of qualitive data will be done using thematic analysis. Based on the data analysis and synthesis of the findings, an implementation framework will be developed to guide development, testing and scale up of a contextually relevant intervention for perinatal mental health.

**Discussion:**

The situational analysis will help to establish relationships with all relevant stakeholders, clarify the context and hypotheses for the pilot intervention and implementation.

## Introduction

Pregnancy and the first year after childbirth are a sensitive period for mothers, with frequent experiences of emotional distress. Globally, up to 20% of women experience mental health problems during pregnancy, and prevalence is significantly higher for women in low- and middle-income countries (LMICs) than those in high-income countries (HICs) (1). In LMICs, the prevalence of specific mental disorders ranges from 19% for perinatal depression (2), to 34% for antenatal anxiety and 26% for postnatal anxiety (3). A recent systematic review among women in India found that the overall pooled estimate of the prevalence of antenatal common mental disorders (CMDs) was around 22% (4).

Particularly vulnerable are women with a history of previous mental health conditions, and those exposed to synergistic health problems within the context of persistent social and economic inequalities (as described in the concept of syndemics) (5), such as poverty or intimate partner violence and crises of health and safety (6–8). Across India, lower socioeconomic status, poor education, unemployment, and bad relations with in-laws are correlated with mental health conditions in perinatal women (9, 10). In rural India, a study in perinatal mothers found that predictors of maternal psychological distress were infant loss, an unwanted pregnancy, health problems and socioeconomic disadvantage (11). Additional risk factors for maternal depression found across India, are having a previous girl child, the desire or pressure to have a male child, and the birth of a female baby (12, 13).

Suicide has been noted to be a leading cause of death in the perinatal period (14), in LMICs particularly young women aged 15–19 years are at risk (15). Even though pregnancy is generally thought to have a protective effect during this period against suicide for mothers, it was found that this was reduced during and after pregnancy for women below the age of 20 years, or those where a pregnancy ends in stillbirth, miscarriage, abortion, or was unwanted (16). In India, specific socio-cultural factors influence the risk for suicide in perinatal women. For instance, increased risks may arise due to infertility, or in relation to unlawful, but still widely practiced, gender birth control methods in order to obtain an often-preferred male child (16, 17). Suicidality amongst antepartum mothers in early pregnancy (<20 weeks) was found to be 7.6% in a sample from South India (18). The study also found that depression severity and a history of suicidal ideation were the strongest predictors of suicide and calls for urgent mental health assessment in women during pregnancy.

Perinatal mental health conditions are correlated with physical disorders, such as gestational hypertension and preeclampsia (19, 20), gestational diabetes (21, 22), preterm birth (23, 24), miscarriage (25), and chronic physical health conditions (26). Conversely, perinatal mental health problems can also lead to difficulties in maternal selfcare, social relationships, and in challenges in infant bonding, breastfeeding and bringing up the child (27, 28).

Perinatal mental health problems can severely affect the infant before and after birth. Perinatal mental health problems correlate with low infant birth weight, restricted foetal growth, as well as social, emotional, behavioural and cognitive development of the child and changes in brain structures and functioning of the child (29, 30). For instance, higher perinatal depressive symptoms have been found to lead to premature brain development in children, including reduced thickness and diffusivity of their brains (31).

Stigma and discrimination are key contributors of distress and harm experienced by people living with mental health conditions, which are inhibiting help-seeking, access to care, and the effectiveness and continuity of treatment. In India, women living with mental health problems during pregnancy are frequently experiencing stigma and discrimination (32), and, in addition, they are often facing intersectional stigma due to their lower status in society (33).

In recent years, mental health and perinatal mental health have gained more attention globally. However, research evidence on the prevalence of perinatal mental health and effective interventions in LMICs generally, and India specifically, remains scarce (34–36). In India, two meta-analyses estimated that pooled prevalence of antenatal depression was 17.7% (37) and 22% and for postpartum depression (38). Evidence on perinatal anxiety in LMICs is rare, only one recent meta-analysis found that the pooled prevalence of self-reported antenatal anxiety was 29.2% and 24.4% for postnatal anxiety, while the prevalence of clinically-diagnosed anxiety disorder was 8.1% antenatally and 16.0% postnatally (39). The evidence gap on perinatal mental health in most LMIC remains substantial, particularly in the lowest income countries, and calls have been made for more research, to better support women in this critical period of their and their children's lives (1, 39). More recently, a few studies started to explore women's antenatal and postnatal mental health in India and developing culturally appropriate measurement tools (1, 40), however they stress that there remains a substantial gap in screening of perinatal women for mental health, and of culturally appropriate, locally validated interventions to address PMDs. Other studies have trialled digital interventions across several states in India which aim to also alleviate the workload from community health care workers (41, 42), however they were not targeting women's mental health, and reach is limited as mobile phones are only available to around 18% of women in rural areas. Recently, the World Health Organisation (WHO) has published a comprehensive guide for integrating mental health into maternal and child health services (43), and guidelines for providing mental health care in community settings are available (27, 44). Peer-delivered, low-intensity psychological intervention could be a cost-effective and potentially effective intervention to support women in their perinatal mental health (45), however there is no evidence for perinatal mental health group interventions in India. In summary, it is not known which interventions are effective, feasible and acceptable to support women who are experiencing mental health conditions during pregnancy and in the year after birth in their communities in rural India.

The SMARThealth Pregnancy (SHP) study is a large 4-year cluster randomised trial (funded by UKRI, see also https://www.wrh.ox.ac.uk/research/smarthealth-pregnancy-improving-women2019s-life-long-health-in-rural-india), across two districts in Telangana and Haryana aiming to reduce anaemia, diabetes, and hypertension in the last trimester and in the first year after birth and identify women at highest risk of longer-term cardiovascular complications. SMARThealth Pregnancy, has been developed to improve the screening, diagnosis, management and postnatal follow-up of women with high-risk pregnancy conditions living in rural India (46). The technology uses the George Institute's SMARThealth platform, extensively tested in India, Indonesia, Thailand, China, and Australia (47–49). The SMARThealth technology has also been applied to mental health in populations in India (50). The platform trains and equips community health workers with screening tools for high-risk conditions, with a decision support algorithm and referral guide based on local guidelines and best practices. The platform facilitates follow-up visits based on the priority of the patient. A primary care doctor App within the platform is paired with the community health worker app, facilitating bi-directional communication. The doctor app also contains prescribing guidelines based on local guidelines and best evidence. The system creates a secure electronic health record and allows for data analytics at the provider, local and district level. SMARThealth Pregnancy presents a unique opportunity for incorporating a perinatal mental health intervention. Before introduction however, it is important for such an intervention to be co-designed and relevant to the wider policy, health system and cultural context before it can be tested and introduced into a specific setting.

We therefore aim to understand the situation and context of perinatal mental illness in rural India. This will be a first critical step in assessing and developing targeted interventions to support women experiencing mental health conditions during their perinatal period. In this study, context refers to concepts used in global mental health (51), including but not limited to, formal and informal perinatal mental health and care systems and services, policies and legislation, social and political context, and cultural values and norms, as well as stigma and discrimination. In addition, we aim to understand what relevant cultural values and norms in relation to perinatal mental health exist in communities in rural India, and to assess the factors, barriers, and facilitators, including stigma and discrimination.

This study will help to understand the situation and context of perinatal mental health for women living in rural India. To start with, we will focus on the state of Telangana where the SMARThealth Pregnancy intervention is currently being tested, with the aim of identifying current barriers, facilitators, and gaps so as to inform development of a future perinatal mental health intervention, which can then be adapted and scaled up more widely across India.

### Aims

The primary aim of the SMARThealth PRegnancy And Mental Health (PRAMH) study is to understand the context of, and assess facilitators, barriers, and gaps for perinatal mental health, in India, specifically in Telangana. The secondary aims are to establish relationships and collaborations with key stakeholders in the field, specifically including people with lived experience, and to develop a synthesis and generate hypotheses for the design and implementation of a new perinatal mental health intervention to be incorporated into the SMARThealth Pregnancy program.

## Methods

### Study design

We will conduct a cross-sectional situational analysis on perinatal mental health using both primary and secondary data. Situational analysis is a suggested methodology for analysing complex systems, such as health systems, and their contexts (52). The method has been previously successfully applied to perinatal mental health (53). This study will be primarily using qualitative methods.

As a first part of the situational analysis, a desktop and literature review will be conducted to assess the sociodemographic and economic context, maternal health and mental health policies and legislation, and perinatal mental health policies and plans. Secondly, interviews and focus group discussions will be carried out to understand gaps identified from the literature review and understand barriers and challenges in implementation. Thirdly, a theory of change workshop with key stakeholders will be conducted to synthesise the findings.

### Study setting

This qualitative study will be conducted in India, specifically in the state of Telangana, in South India. Telangana is a relatively new state in South India (after separating from Andhra Pradesh in 2014) with a total population of 35 million (as per 2011 census), 60% of whom are living in rural areas (54). The maternal mortality rate in Telangana has been reported in the periodic sample registration system (SRS) 2016–2018 as 63 per 100.000 live births, which is comparably low to the national average of 113 (55).

The study setting has been chosen as this is a site in which the SMARThealth Pregnancy trial is currently being run, and thus we have established relationships with ASHA workers, primary care centre staff, and district medical officials. The site is suitable as it has a high prevalence of mental health conditions compared to the national average, for instance for depression, the ratio of Telangana's state Disability Adjusted Life Years (DALY) to median DALY rate for all states is 756 (527–1025), compared to 550 (390–748) for India (56). For anxiety, the rate for Telangana DALY is 324 (228–434), compared to 309 (220–414) for India. Telangana has a developing health system (57), and cultural and gender context and challenges, which can be seen as representative for India (58).

Traditionally a 6-week post-partum period is considered as the postnatal phase, but in order to capture late maternal deaths and to prevent ill health and promote long-term physical and mental health, it is more appropriate to understand the period until up to 1 year after birth as the postnatal period (59).

### Sampling

Participants of the study will be selected through purposeful sampling, using key experts already involved in the SMARThealth Pregnancy programme and pursuing a snowballing approach. In addition, further key experts in the field will be identified and included as participants throughout the completion of the situational analysis tool.

### Participants

Key stakeholders related to rural and semi-rural primary health care (PHC) centres from Siddipet district (Telangana) will be selected to participate in the study, based on either their background in perinatal mental health, interest to engage with the PRAMH study, or role in providing primary health care. Purposive selection of two PHCs will be done, to represent both urban and rural communities. Key stakeholders will include women with lived experience of perinatal mental health problems, community health workers, known as Accredited Social Health Activists (ASHAs), Auxiliary Nurse Midwives (ANMs) and Anganwadi Workers (AWWs), as well as district medical officers, psychiatrists, and psychologists.

### Recruitment and consent

Participants will be identified through the situational analysis, existing personal contacts, and snowballing. Specifically, contacts and stakeholders from the SHP study will be contacted and invited if they qualify for the PRAMH study. Eligible participants will be women with lived experience of perinatal mental health problems, who will be identified by ASHAs and ANMs. Women with lived experience will also be approached through the SHP2 study, if they score highly on the baseline PHQ-9 or GAD-7 or report a mental health history in the screening. We will also invite the women's families/carers, perinatal and mental health care professionals and community health workers, including ASHAs, ANMs, and Anganwadi Workers (AWWs). In addition, policymakers, planners, state and district level health officials, mid-level providers of the new health and wellness centers (HWCs), panchayat village heads, mental health specialists, primary care providers, maternal healthcare providers, AYUSH (Ayurveda, Yoga & Naturopathy, Unani, Siddha and Homoeopathy) doctors, nurses, and civil society organisations, will be invited to participate.

Participants for the interviews and focus groups will be recruited through contacts and the existing network of the SHP study (ASHAs, ANMs, health care providers, researchers, etc., in Telangana). We will work with the participating PHCs and district health authorities before commencement of the study to strengthen relationships. See Table 1 for details.

**Table 1:** Overview of recruitment of key stakeholders in Telangana.

Key stakeholder	Method of data collection	*n*	How they will be approached
Policymakers and planners			
Policymaker	In-depth interview	1–2	The study teams and their contacts will reach out to people that are known to them through the SMARThealth Pregnancy study and through the wider network of the George Institute in India; additional relevant participants will also be identified through the scoping review.
District health official/planner	In-depth interview	1–2	
Panchayat village head	In-depth interview	1–2	
Mid-level provider	In-depth interview	1–2	
Health professionals			
Psychiatrist	In-depth interview	1–2	
Psychologist	In-depth interview	1–2	
Obstetrician/gynaecologist	In-depth interview	1–2	
PHC doctor	In-depth interview	1–2	
AYUSH doctor	In-depth interview	1–2	
ASHAs	Focus group discussion	10–15	
Anganwadi workers, ANMs, nurses	Focus group discussion	10–15	
People with lived experience and community			
Women with lived experience	Focus group discussion	10–15	The women will be identified and approached by the ASHAs and ANMs affiliated with each PHC, and through the SHP2 study, if they score high on the baseline PHQ-9 or GAD-7 or report a mental health history in the screening.
Family members, care givers, community members	Focus group discussion	10–15	The women's families, caregivers, and community members will be identified by the ASHAs and ANMs affiliated with each PHC.
Civil society organisation	In-depth interview	1–2	The study teams and/or their contacts will reach out to people that are known to them through the SMARThealth Pregnancy study and through the wider network of the George Institute in India; additional relevant participants will also be identified through the scoping review.
Women finance self-help group lead	In-depth interview	1–2	
	Total *n*	50–80	

This study will exclude people who decline participation; people who do not speak and understand verbal communication in English or the local vernacular language (Telugu); and people below 18 years of age.

Informed written consent will be obtained from all participants by a member of the study team prior to the interviews.

### Outcome measures

The expected outcomes for the PRAMH study are:

• Outcome 1 (objective 1): A completed situational analysis tool, which will clarify perinatal mental health needs in the study setting, context, mental health policies and plans, mental health treatment coverage, district level health services, community setting, and monitoring and evaluation plans.
• Outcome (objective 2): Learnings on perinatal mental health in rural Telangana, specifically in people's knowledge, beliefs, and attitudes, and in regard to context and implementation challenges and barriers of perinatal mental health assessment and intervention.
• Outcome (objective 3): An implementation framework specifying the defined priorities, hypotheses, design and outcomes for a new future perinatal mental health intervention in rural Telangana.

## The SMARThealth Pregnancy And Mental Health (PRAMH) situational analysis

The PRAMH study will be composed of three components.

(1) Completion of situational analysis tool, using a desktop and literature review.
(2) Analysis of context, barriers and facilitators in relation to perinatal mental health and related interventions, using in-depth interviews and focus group discussions
(3) Development of a framework to guide a future intervention and implementation in the next phase of the study, using a set of theory of change workshops.

**Figure 1:**
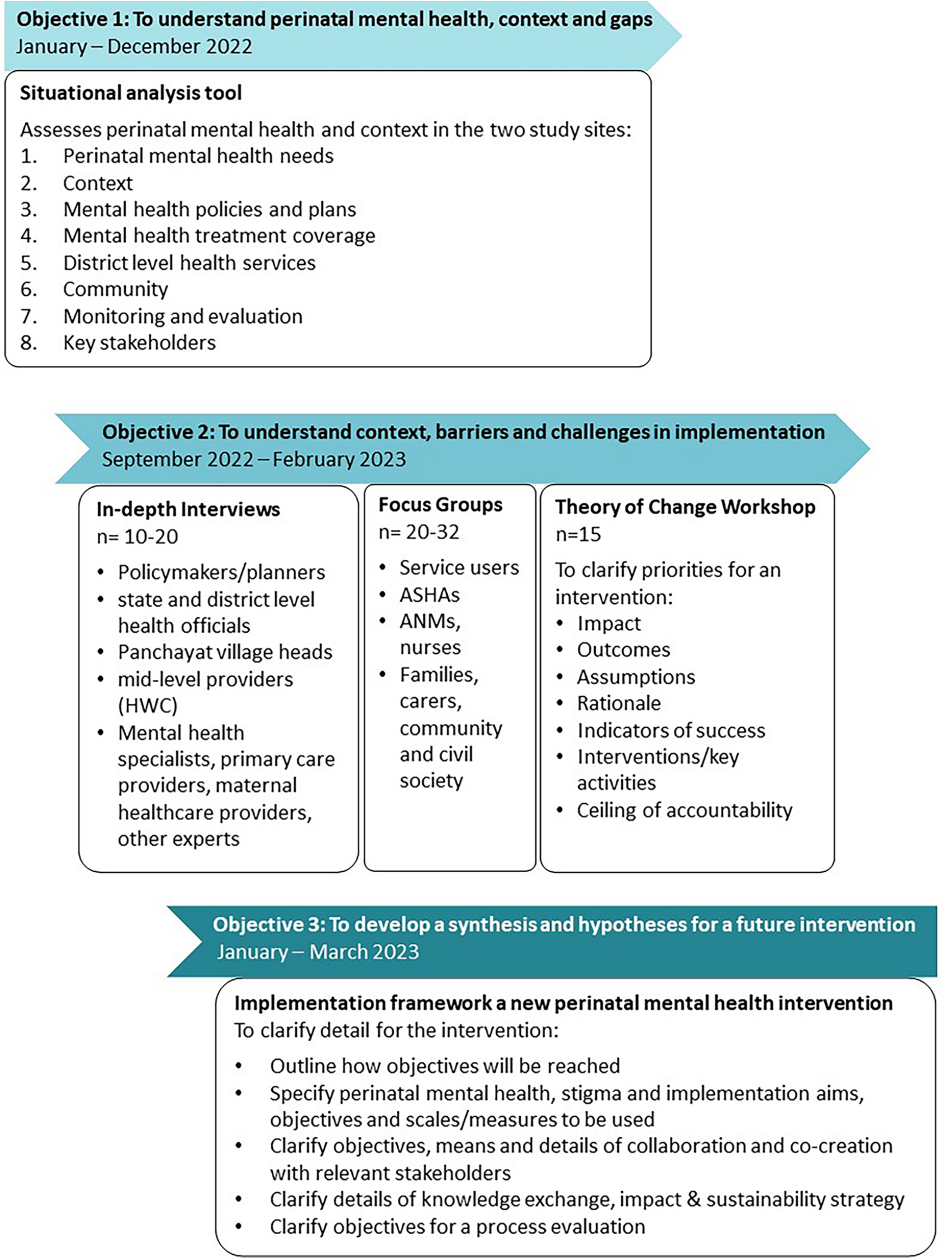
PRAMH study schema of formative and intervention development.

Figure 1 outlines the study schema of formative and intervention development.

### Situational analysis tool

We will use a standardised situational analysis tool, adapted from the PRIME study/WHO-AIMS (53, 60). The situational analysis will begin with a comprehensive desktop and literature review to assess perinatal mental health, policies and initiatives at the country and state level. This will be complemented by a second phase involving in-depth interviews and focus groups to understand the current situation for women with perinatal mental health problems in selected rural settings.

The situational analysis will investigate seven themes, exploring context, (perinatal) mental health policies and plans, treatment coverage, district-level health services, community, monitoring and evaluation, key stakeholders.

Sources of data for the desktop review will include web pages, publicly available reports and other documents from state and national Ministries of Health and Welfare, the National Institute of Mental Health and Neurosciences (NIMHANS), National Family and Health Survey (NFHS) health surveillance reports, peer-reviewed research publications, and will be supplemented by personal communication with experts in the field.

### In-depth interviews

In-depth interviews (IDIs) will be conducted to understand local stakeholders’ perspectives on perinatal mental health and perinatal mental health conditions, existing approaches to cope with mental distress, as well as acceptability and feasibility of brief psychological interventions, and cultural and structural issues related to the implementation of the intervention. The design of the topic guides for the IDIs and FGDs will be informed by the themes of the situational analysis and concepts from Proctor et al.'s taxonomy of implementation science (61).

The IDIs will be conducted in Telangana with policymakers and planners, health professionals, and civil society members (see Table 1 above). IDIs will be also conducted for women and family members with lived experience who for reasons of health condition, stigma and discrimination, or other reasons, are unable to participate in focus group discussions. It is estimated that approximately 5–10 IDIs will be conducted in total (*n* = 10–20).

Members of the research team will be trained by the study lead in conducting qualitative interviews. The interviews will be conducted using a semi-structured interview guide. The in-depth interview topic guides will be informed by Kleinman's explanatory model interview (62, 63). Other studies have used qualitative interviews to assess barriers to women seeking and receiving help for perinatal mental health problems and developed questionnaires (64) and perspectives on antenatal mental health problems and to assess the potential for psychological interventions (65).

### Focus group discussions

The focus groups will aim to identify concepts and contexts of perinatal mental health problems, existing approaches to cope with mental distress, as well as acceptability and feasibility of brief psychological interventions, and cultural and structural issues related to the implementation of the intervention. Focus groups will be organised with community health workers and community members, in order to provide a safe, stress-reduced environment for all participants, respecting cultural, gender and power differences. From each stakeholder group, it is expected that 5–8 representatives will participate in 1–2 FGDs, thus in total 20–32 participants (see Table 2).

**Table 2:** Overview of focus group discussions (FGDs).

Participants	Number of FGDs	*N* per FGD
Women with lived experience	1–2	5–8
Family members, care givers, community members	1–2	5–8
ASHAs	1–2	5–8
Anganwadi workers, ANMs, nurses	1–2	5–8
Total	4–8	20–32

Members of the research team will be trained by the study lead in facilitating focus group discussions. The FGDs will be conducted by the trained local research assistant and a local assistant/note taker, who speak the local language (Telugu), allowing for the smallest power grade between researcher and participants and low barriers for conversational norms (66). FGDs with women with lived experience will be carried out by female researcher/assistants only.

### Theory of change

A theory of change (ToC) process will be conducted as a priority setting exercise and to clarify challenges and opportunities, priorities, and objectives to inform development of a future intervention. ToC is an established, theory-driven approach to co-produce, plan and evaluate programmes in global mental health and beyond (67–69).

The ToC will include relevant stakeholders and will be developed using a stepwise approach:

(1) Based on the results of the PRAMH formative work, and using an established step-by-step guide (70), the research team will map out a draft ToC, including the hypothesised causal pathways, stakeholders, key outcomes, and intervention/s necessary to achieve the health service outcomes and the ultimate impact of the PRAMH study (improved health, social and economic outcomes for women experiencing perinatal mental health problems and their families/carers).
(2) After completion of the interviews and focus group discussions, a ToC workshop will be conducted with selected relevant stakeholders (estimated *n* = 15), to identify feasible intervention(s), human and other resources required, contextual barriers and facilitators for implementation and outcome and impact indicators. The main aims will be: (1) to clarify the priorities and hypotheses, specifically the impact, outcomes, assumptions, rationale, indicators of success, interventions/key activities and ceiling of accountability (67); (2) to introduce the team, build relationships and engage stakeholders, and to ensure stakeholder buy-in and bottom–up development of a new, future perinatal mental health intervention (71).
(3) A checklist will be used to report the ToC approach (72).

### Outcomes and materials

Based on the data analysis and synthesis of these findings and drawing on Proctor et al.'s implementation taxonomy (61), an implementation framework will be developed to inform an intervention strategy for a new perinatal mental health intervention. In addition, the framework will help clarify the objectives for a process evaluation. A network collaboration map will be developed which will outline the objectives and details for collaboration and co-creation with key stakeholders including patient and public involvement (PPI) representatives and people (women) with lived experience of mental health conditions (PWLE). A knowledge exchange, sustainability and impact roadmap will be developed, which will clarify means and details of knowledge exchange and impact strategy. A training package will be developed containing information on perinatal mental health conditions, their management, and their long-term implications on women's health, as well as stigma and discrimination. A context and culturally sensitive, equity and gender-based approach will be taken to co-creation and data collection with people with perinatal mental health problems.

### Data collection

Independent, trained field investigators will be involved in data collection throughout the study. Data collection will be cross-sectional, taking place between August and December 2022. All interview and focus group data will be recorded and transcribed and translated. Data will be de-identified, saved and stored in a secure server.

The PRAMH study is nested within the wider SMARThealth Pregnancy study (www.ClinicalTrials.gov, identifier: will be added) and may use some of the baseline screening to understand the context, adapt the scales, assess implication of the overall study, and to help power future interventional studies.

### Detection of new medical complications during the study

If any of the participants are experiencing or are suspected of having a new perinatal health problem that they have not received treatment for, they will be supported by the study team to be referred based on existing referral pathways.

The study team will be trained to recognise women who are potentially victims of domestic violence If a case of domestic violence is suspected, they will report it to a senior researcher/project supervisor, who will then identify safe ways of talking to the woman and suggest options that can be taken to initiate action to support her. If during the study, any of the participants reveal, or are suspected to be experiencing domestic violence, this will also be appropriately escalated. If the woman agrees, the senior researcher will get in touch with an identified, safe, civil society organisation to take over the support.

### Qualitative analysis

A qualitative, thematic analysis will be performed on the primary data. The interviews and focus group discussions will be transcribed and translated into English and checked by a speaker fluent in both languages. They will then be coded using NVivo software and a thematic analysis/framework analysis will be performed, using deductive analysis, i.e., pre-defined themes that were defined and earlier used and obtained from the situational analysis tool. The themes and sub-themes will be supplemented by inductive coding, and new themes will be added as they emerge from the data. The final analysis is anticipated to be competed 6 months after data collection.

### Theoretical framework

The PRAMH study has several theoretical underpinnings. The research approach taken will be through a post-positivist and constructivist lens [consensual qualitative research (CQR)] (73). The critical realism/relativist ontology will assume that an approximal reality and general core ideas can be agreed upon, while recognising varying experience of individuals contributing to the data collection.

The study uses a largely qualitative approach for data collection, and a predominantly deductive approach for data analysis. Implementation science concepts will inform data collection (the IDI and FGD topic guides), data analysis, guide the ToC, and the development of the framework, namely Proctor et al.'s eight conceptually distinct implementation influences: acceptability, adoption, appropriateness, feasibility, fidelity, implementation cost, penetration, and sustainability (61). Framework analysis will be used to develop the new implementation framework.

### PPI co-creation

Experts by experience, otherwise known as people with lived experience (PWLE), and their families and carers are involved as key contributors of this study, in order to ensure a co-creational approach to data collection and analysis. Service user informed research involvement is key to developing and implementing sustainable, effective mental health interventions. A systematic effort is being undertaken to identify and involve PWLE (including through the ongoing SMARThealth Pregnancy trial), who are interested in contributing. The Global Mental Health Peer network has been contacted for PWLE and a snowballing approach is being used to involve experts by experience.

### Data monitoring and confidentiality

Data collected during the PRAMH study will be securely uploaded and stored on a secure server at the George Institute, India. All data will be de-identified and anonymised. Data will be monitored for quality and completeness by the programme manager at regular intervals as it is uploaded to the server. All data will be stored and handled in accordance with the principles of the UK Data protection Act and the Indian 1998 Data Protection Act.

### Ethical approval

This study has approvals from the University of Oxford Tropical Research Ethics Committee (OXTREC reference number 539-22) and the George Institute for Global Health India Institutional Ethics Committee (IEC; reference: 09/2022).

A study steering committee will be comprised and oversee the study and be responsible for participant safety. Regular operation group meetings and field team meetings will be held with relevant members of the team. The study sponsor is the George Institute for Global Health, and the study will be funded by the UKRI Future Leaders Fellowship (awarded to JH).

## Discussion

The situation for women experiencing mental health problems during their pregnancy and postpartum in rural India is critical. Perinatal mental health problems have a high prevalence and often go unrecognised and untreated in rural India, while at the same time there is a lack of empirical studies. This situational analysis will explore the context of, health systems and services, policies, barriers, and facilitators for, perinatal mental health in India, and specifically in the state of Telangana. A desktop review will be conducted to review existing literature on perinatal mental health, followed by in-depth interviews and focus group discussions, and a theory of change workshop, which will develop an implementation framework for a future intervention to assess perinatal mental health and support women experiencing perinatal mental health problems.

Perinatal mental disorders have been classified as significant complications of women's pregnancy and postpartum periods (74). Women in their perinatal period frequently experience poor mental health, which exist along a continuum ranging from mild, time limited psychological distress, to chronic, progressive, and severely disabling conditions. While psychological distress is frequently associated with pregnancy and the changes in the postnatal period, it is usually time limited. It can however result in reduced social functioning, and self-isolation. Mental health conditions, in the medical discourse often referred to as “disorders”, are marked by clinically significant changes in thoughts, perceptions, emotions, or behaviour, and are frequently associated with distress or impairment in functioning or risk of self-harm (75, 76). They cause significant functional impairment and affect relationships with other people and the ability to accomplish expected social roles. Further, they also have a significant association with negative outcomes in the emotional, sociopsychological and behavioural development of infants and children of women experiencing mental health conditions in the perinatal period (30). Psychosocial disability describes the interaction of mental impairments and social barriers to participation in society, which women with mental health conditions frequently experience (social model of disability) (77).

In this study, we are referring to mental ill health most common in perinatal women, namely psychological distress, social isolation, depression, anxiety, psychosis, and suicidal ideation. While postpartum blues (“baby blues”) is very common in postpartum women, it is usually temporary and subsides after 7–10 days, and more effectively so if social support, good relationships, and care are in place. However, it is a risk factor for developing postnatal depression and anxiety, particularly under adverse conditions (78). Screening for perinatal mental health problems in LMICs can be challenging and it is relevant that tools are locally validated and culturally appropriate to ensure women with mental health problems can be identified (1).

### Stigma and discrimination

Stigma and discrimination are critical factors that are limiting the impact of health interventions for people with mental health problems and disabilities, and they are linked to underdiagnosis and exclusion from access to care (79, 80). Stigma and discrimination reinforce inequalities, are heightened in conditions of intersectionality, such as gender/race, and therefore affect women in LMICs disproportionately (33, 81).

Across India, stigma and discrimination against people with mental health conditions is widespread (82), and suicide is a highly stigmatised topic (15).Women living in deprived socio-economic conditions, those with low societal status, as well as those experiencing intimate partner violence are at a greatly increased risk of perinatal mental health conditions (1).

The recent Lancet Commission on Ending Stigma & Discrimination in Mental Health 2020–2022 highlights the current evidence and effective interventions for reducing stigma, and provides guidance for global action, interventions and policy change in low- and middle-income countries (83). Co-creation and active, non-tokenistic involvement of people with lived experience is a key facilitator for addressing stigma, reducing discrimination and for making global health research programmes more inclusive (84). In particular, social contact has been found to be the most effective ingredient for reducing stigma (85).

Stigma and resistance are likely to be challenges, not only in relation to the women but also with other stakeholders such as health care workers and community. Awareness, knowledge and skills around perinatal mental health conditions and their treatment are expected to be limited in rural India, even for health care workers and professionals (86). Throughout the PRAMH study, careful consideration will be given to assess and address stigma and discrimination throughout design, development, and implementation of the intervention, including capacity building for perinatal mental health. Building on concurrent research and collaborations with colleagues, such as the Indigo Programme (80, 85), or the SMART Mental Health study (50, 87), the components of the PRAMH study will be co-created with people with lived experience, health workers, policymakers, and other stakeholders.

### Limitations

In relation to data collection, getting access to women with lived experience may be a challenge. In Indian rural areas, women with mental health experience are often sent to their paternal residence by their spouses/in-laws for extended periods, which may influence access to these women and their realities. The varied ways of data collection with various stakeholder groups, including a revision of the framework through theory of change workshop should help to maximise the understanding of the situation.

Some of the participants will be recruited via the SMARThealth Pregnancy study where women with anaemia, hypertension and diabetes are screened, diagnosed, and managed. These physical health conditions might in themselves pose a risk for various mental health problems specially related to perinatal period, and equally may lead to somatic symptoms which can overlap with symptoms of mental ill health. For instance, anaemia is associated with an increased risk of perinatal depression (88), and may be associated with anxiety (89, 90).

A potential future challenge when implementing this study may be related to attrition and preventing dropouts from the study. Traditionally, many pregnant women travel to their parental residence/village during the last phase of their pregnancy and stay there until a few weeks or months after birth, which may cause dropouts of the study. This is particularly the case for the women's first pregnancy and may be different for subsequent pregnancies. Further, some mental health problems may arise specifically in relation to the first pregnancy, such as the stress and anxieties related to the persistent gender bias and pressure of having a boy child, or the unmasking of existing mental health problems during that phase.

Over the past 20 years, great progress has been made in India in reducing maternal mortality, and a priority now lies in reducing maternal morbidity arising from mental health conditions (38). There is great potential for perinatal mental health care to be part of a lifelong integrated approach to health care delivery. We have plans for a future validation in other sites in India, in particular in Haryana, where the SHP2 trial is already running.

## Conclusion

This protocol outlines the aims and objectives of a situational analysis study of the PRAMH study, with a view to co-creating an intervention suitable for women living in rural India. The situational analysis will help to establish relationships with all relevant stakeholders, clarify the context and hypotheses for a subsequent pilot intervention and implementation. More broadly, this study will help establish and summarise the evidence base on women's mental health during the perinatal period in rural India and highlight opportunities for potential interventions and care pathways to address perinatal mental disorders.

## Ethics statement

This study has approvals from the University of Oxford Tropical Research Ethics Committee (OXTREC reference number 539-22) and the George Institute for Global Health India Institutional Ethics Committee (IEC; reference: 09/2022). The patients/participants provided their written informed consent to participate in this study.
